# Juntos: A Model for Language Congruent Care to Better Serve Spanish-Speaking Patients with COVID-19

**DOI:** 10.1089/heq.2020.0124

**Published:** 2021-12-08

**Authors:** Santiago Alvarez-Arango, Tina Tolson, Amy M. Knight, Sophie K. Presny, Dulce M. Cruz-Oliver, Sabrina Aloe, Jessica Contreras, Nicole Dzamko, Adrianna Moore, Inez Stewart, Sherita H. Golden, Kathleen R. Page

**Affiliations:** ^1^ Johns Hopkins University School of Medicine, Baltimore, Maryland, USA.; ^2^ Johns Hopkins Health System, Baltimore, Maryland, USA.

**Keywords:** COVID-19, Latino community, limited English proficiency, cultural-based communication, minority tax

## Abstract

Coronavirus disease 2019 (COVID-19) exacerbated pre-existing health disparities and disproportionately affected the Latino community. Clinicians identified communication barriers as a major challenge in care for COVID-19 Latino patients with limited English proficiency (LEP). To address these challenges, Juntos (Together) consult service was established to promote language-congruent care with cultural sensitivity, identify barriers to safe discharge, and facilitate referral to appropriate resources. Spanish speaking volunteer health care providers worked synergistically with medical teams caring for LEP Latino patients. Volunteers were trained on consultant responsibilities and discharge planning resources. The program was evaluated by a satisfaction survey distributed to providers who requested a Juntos consult and Juntos volunteers. Between May 5 and July 30, 2020, 19 individuals volunteered time to the Juntos consult service, 12 (63%) Latinos, 14 (74%) physicians, and 5 (26%) staff. The service supported 127 patients, 76 (60%) males, mean age 42 (±16), 83 (65%) uninsured, and 91 (72%) without primary care. The most common referral sources were medical units (52, 41%) and intensive care units (47, 37%). The most common services offered were family engagement (55, 43%), goals of care (35, 28%), and mental status assessment (26, 20%). The majority of providers who consulted Juntos were very satisfied (48/59, 81%) with the care delivered. The Juntos service offered critical support tailored to the patients' and primary teams' needs. The experience reinforced the need for cultural-based communication to provide optimal care to LEP patients. The Juntos consult service could be a model for providing language-congruent care even beyond COVID-19, but to do so will require institutional investment and rigorous outcomes evaluation.

## Background

The coronavirus disease 2019 (COVID-19) has exacerbated pre-existing health disparities and disproportionately affected the Latino community.^1–5^ In the United States, the rate of COVID-19 is more than three times higher among Latinos than in non-Hispanic whites (73 vs. 23/10,000), and the age-adjusted hospitalization rate more than four times higher (160.7 vs. 40.1/100,000).^6^ The disparities have been particularly stark among younger individuals. For example, 34.9% of Latino individuals who died of COVID-19 were <65 years of age, compared to 13.2% of non-Hispanic whites.^7^ Almost half (44.6%) of COVID-19 patients <21 years of age are Hispanic.^8^

At the Johns Hopkins Health System (JHHS), these disparities became readily apparent early in the epidemic. Between March 11 and May 25, 2020, the SARS-CoV2 positivity rate was 43% among Latinos, compared to 18% among blacks and 9% among whites.^4^ The majority (66%) of Latino patients admitted to JHHS with COVID-19 were Spanish speakers with limited English proficiency (LEP). Facing an unprecedented number of Spanish-speaking Latino patients admitted with COVID-19, a group of clinicians convened a working group with the Johns Hopkins Vice President of Diversity, Inclusion and Health Equity (S.H.G.), the Vice President of Human Resources (I.S.), the Director of Language Access Services (T.T.), and Centro SOL leadership (K.R.P.) to develop and implement a strategic response.^9,10^

Anecdotally, Johns Hopkins Medicine (JHM) clinicians identified communication barriers as a major challenge when caring for COVID-19 patients with LEP. In-person interpretation services were limited, and third-party remote interpretation was challenging across layers of personal protective equipment. In addition, clinicians felt ill-equipped to address health literacy and cultural nuances during complex patient and family conversations or to provide adequate support for immigrant patients' psychosocial needs.

To address these challenges, the Juntos consult service was established. “Juntos” means “together” in Spanish. The service was staffed by volunteer bilingual clinicians and social workers who worked synergistically with the medical teams to explain the treatment plan to patients and relatives, explore the patients' social context, and facilitate postdischarge care.^11^ The purpose of this article is to describe the Juntos implementation process and outcomes below.

## Methods

### Identification of qualified bilingual staff

In May 2020, as the profound impact of COVID-19 among Spanish speaking Latinos became readily apparent, the JHM Office of Diversity, Inclusion, and Health Equity and JHM Language Services conducted a system-wide survey to credentialed providers asking about (1) non-English language proficiency, (2) language certification, (3) hospital affiliation, (4) clinical position, and (5) willingness to be deployed to COVID-19 units (Supplementary Data S1). Bilingual providers who had not completed the qualified bilingual staff (QBS) assessment were strongly encouraged to do so, with the testing fees paid by the JHHS system during this emergency.

### Juntos: bilingual clinicians supporting patients “together” with the clinical team

**FIG. 1.**
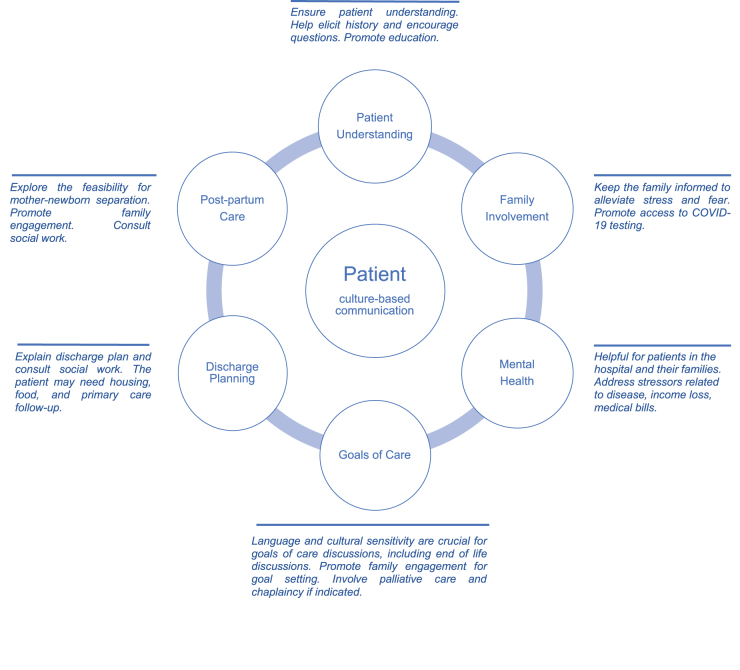
Patient-centered Juntos Model of Care.

Juntos was established to help provide equitable resources and strategies to LEP Latino patients with COVID-19 admitted to the Johns Hopkins Hospital (JHH) or the Johns Hopkins Bayview Medical Center (JHBMC), academic-based hospitals in an urban setting. The overall goal of this service was to work synergistically with the medical, clinical, and social work teams to (1) promote language-congruent care with cultural and literacy level sensitivity; (2) identify barriers to safe discharge, with attention to issues of public health concern (e.g., crowded housing or work conditions, access to food); and (3) advocate and facilitate referral to appropriate resources needed to support the patients and their communities (Fig. 1). The service was not designed to replace medical interpreters or the need for a primary social worker and case manager.

The Juntos Consult Service officially launched at JHH and JHBMC on May 5, 2020 and was announced through a system-wide email from the Office of Diversity, Inclusion, Health Equity, and JHM Language Services. A channel named “Juntos” was established for clinical teams to request services in the homegrown paging system. Consults could be requested by anyone on the clinical or social work team caring for Spanish LEP patients who had been admitted with COVID-19. Because of limited capacity, Juntos did not cover the Emergency Department. During the peak of COVID-19 admissions (May 5–July 30), consultants were available 7 days a week from 7 a.m. to 7 p.m. As COVID-19 admissions declined and volunteers returned to their pre-COVID-19 responsibilities, consults were staffed from Monday through Friday 8 a.m. to 5 p.m.

### Resources and training for Juntos Providers

Before the program pilot's formal launch on May 5, 2020, volunteers were trained on their roles and responsibilities. A Juntos tip sheet (Supplementary Data S1) was developed, updated through iterative feedback, and distributed to consultants outlining their roles and responsibilities. The tip sheet included the Juntos mission statement, consultant roles and responsibilities, discharge planning resources (including primary care options for patients ineligible for insurance coverage through the Affordable Care Act), legal resources for immigrants, food and housing assistance, and Spanish language palliative care resources for families.^12,13^ The program leaders reached out to community partners, Federally Qualified Health Centers, and the Maryland Department of Health to leverage existing resources (such as food distribution, isolation hotel, i.e., government-sponsored hotel for those unable to isolate at home, etc.) to support patients after discharge. COVID-19 follow-up was conducted via an outpatient telemedicine program staffed by physicians and nurses from JHM.

A Juntos consult service was implemented in the electronic health record (EHR), and a patient list for the service was shared with the volunteers. Emphasis was made on sign-off and transition of care given the volunteer nature of the program. The service also developed a standard note template to provide consistent documentation and highlight the goals outlined above and made Spanish COVID-19 educational materials available in the EHR.

### Program evaluation

Patient demographic data were extracted from our electronic medical record system (Epic Corporation, Verona, WI) using operational reports and manual chart searches. Service quality assurance questions were designed (K.P., S.K.P., T.T.), revised (D.M.C.-O., S.A.-A.), and final surveys were created utilizing the Qualtrics survey platform. A link to access the survey was distributed via email using the participants' organizational addresses queried from the consult line. A service satisfaction survey was distributed to health care providers who requested Juntos consults, and a separate survey assessing the consultant experience was distributed to Juntos volunteers (Supplementary Appendix SA1). Using a mixed-methods approach, each survey contained demographic, multiple-choice, Likert scale, and open-ended questions to facilitate quantitative and qualitative statistical analysis. The aim was to gather the participants' impressions of the Juntos service's value, quantify the provider types and clinical areas engaged with the consultants. This work was deemed quality improvement and exempt by the Johns Hopkins Institutional Review Board.

## Results

### Identification of bilingual staff and QBS certification

Between May 1 and May 31, 2020, 588 providers completed the language proficiency survey conducted by the Office of Diversity, Inclusion and Health Equity and the Office of Language Services. Among all respondents, 386 (65.7%) reported non-English language proficiency. Among 274 respondents who specified language proficiency, 98 (35.8%) spoke Spanish, 24 (8.8%) Mandarin, 22 (8.0%) Arabic, and 130 (47.4%) Other. Only 58 of 241 (24.1%) respondents who reported non-English language proficiency had been qualified through the JHM QBS Program, but the proportion qualified was higher for Spanish speakers (45.9%, 45 of 98). More than half of respondents reporting non-English language proficiency were interested in redeployment to COVID-19 units to assist in the care of LEP patients (51.7%, 122/236 overall, and 51.0%, 50/98, for Spanish speakers).

### Juntos volunteers recruitment and characteristics

The Juntos pilot program was launched on May 5, 2020 with 10 volunteers primarily identified by the Juntos founding members. Regular emails from the Office of Language Services encouraged QBS Spanish speaking clinicians to sign up for volunteer shifts. Between May 5 and July 30, 2020, a total of 19 individuals volunteered their time. Among them, 12 (63.2%) were Latino, 14 (73.7%) were physicians (7 faculty and 7 residents or postdoctoral fellows), and 5 (26.3%) were other staff (three RNs, one physician's assistant, and one licensed clinical social worker). The majority (147/178, 82.6%) of consults were completed by only seven volunteers, of whom six (85.7%) were Latino.

### Juntos consult requests and services provided

From May 5, 2020 to July 30, 2020, the team of bilingual volunteer providers from Juntos was consulted for 127 Latino patients with LEP and performed a total of 178 initial and follow-up consults. The mean age of Juntos patients was 42 years (SD=16, range 1.24–81), and the majority were male (*n*=76/127, 60%), uninsured (*n*=83/127, 65%), and did not have an established Primary Care Physician (PCP) before admission (*n*=91/127, 72%). The most common comorbidities were obesity defined as a body mass index of ≥30 (*n*=45/127, 35%), diabetes (*n*=32/127, 25%), and hypertension (*n*=22, 17%). The most common primary teams that consult the Juntos program were the medical intensive care unit, general medicine and surgical floors, and labor and delivery (Table 1).

**Table 1.** Characteristics of Patients Served by the Juntos Team, May 5 to July 30, 2020

Characteristic	Patient consults (*n*=127), *n* (%)
Age—years (SD)	42 (16.4)
Sex	
Male	76 (59.8)
Female	51 (40.2)
Insurance	
Uninsured or self-pay	83 (65.4)
Medicaid/Medicare	17 (13.4)
Commercial	22 (17.3)
Workers compensation	5 (3.9)
Employment	
Unemployed	56 (44.1)
Construction	28 (22.0)
Services^a^	24 (18.9)
Other or unknown^b^	19 (15.0)
Primary Care Physician	
Yes	36 (28.4)
No	91 (71.6)
Comorbidity	
Obesity	45 (35.4)
Diabetes	32 (25.2)
Hypertension	22 (17.3)
Other^c^	
Primary team service	
Medical-surgical unit	52 (40.9)
Medical intensive care unit	47 (37.0)
Labor and delivery	21 (16.5)
Pediatric unit	7 (5.5)
Patient status	
Alive	118 (93)
Deceased	9 (7)
Type of consult	
Patient understanding	92 (72.4)
Primary care follow-up	71 (55.9)
Family involvement	55 (43.3)
Mental Health	26 (20.5)
Goals of care	24 (18.9)
Postpartum care	19 (15.0)

^a^ Services (e.g., cleaner, cook, driver, babysitter, food delivery, secretary).

^b^ Other employment: None (e.g., pediatric patients) and patients on disability.

^c^ Other comorbidities (e.g., alcohol dependence [*n*=14, 11.0%], hyperlipidemia [*n*=11, 8.7%], liver disease [*n*=8, 6.3%], renal disease [*n*=7, 5.5%], respiratory disease [*n*=6, 4.7%], and cancer [*n*=4, 3.2]).

The Juntos program primarily offered services to overcome patients' cultural, health literacy, and language barriers, but identification of obstacles for a safe discharge (e.g., appropriate PCP follow-up, housing, food access) was addressed in most consults as well. The program offered critical interventions based on the particular patient and primary teams' needs such as family engagement (*n*=55, 43%), goals of care (*n*=35, 28%), mental status assessment (*n*=26, 20%), and mother–newborn separation (*n*=19, 15%) (Fig. 1 and Table 2).

**Table 2.** Services Provided by the Juntos Program Between May 5, 2020, and July 30, 2020

Service provided	Patient consults (*n*=127), *n* (%)
Family involvement	55 (43.3)
Education	
Patient understanding	92 (72.4)
Medical comorbidities	71 (55.9)
Communication barriers	
Mental health	26 (20.5)
Physical^a^	17 (13.4)
Goals of care discussion	
Code status including end-of-life discussions	24 (18.9)
Intubation^b^	8 (6)
Safe discharge	
Primary care	71 (55.9)
Field hospital	24 (18.9)
Postpartum care	
Mother-newborn separation	19 (15.0)

Percentages are based on the total number of patients (*n*=127) followed in that period.

^a^ Physical: postextubating, post-tracheostomy, and personal protective equipment.

^b^ Consult requested by the primary team to talk to a patient about the need for intubation.

### Program evaluation

Between June 28 and August 27, 2020, a survey was distributed to 59 health care providers who requested a Juntos service, and 54 responded (92% response rate). The majority (50/59, 85%) of requesting providers utilized one of the approved medical interpretation modalities before placing a Juntos consult. Provider satisfaction was high (48/59, 81% very satisfied and 9/59, 15% satisfied) with the care delivered by Juntos consultants. Written feedback noted that the program elevated the standards of care for their patients, and several respondents recommended that the service be expanded to non-COVID-19 complex medical cases (Table 3). The majority of providers expressed that they were very likely (52/59, 88%) or likely (5/59, 9%) to recommend the Juntos program to their peers.

**Table 3.** Juntos Pilot Program Feedback Comments

Feedback
“… Our patients have had incredibly complicated courses in the ICU, and our teams have previously struggled to convey the gravity of the patient's health, complexity of discharge planning, and engage in shared decision-making”
“Having a Spanish-speaking provider who can be involved in family communication in a more meaningful way than just providing interpretation services is invaluable”
“Invaluable resource in the ICU”
“Juntos program was invaluable in ensuring the patient's family had a complete understanding of [the patient's] degree of illness and poor trajectory… I strongly believe the Juntos program should become a permanent part of the JHH language service team as they were vital in facilitating very complicated and nuanced conversations”
“Excellent program, would love it if this could be continued as a permanent service.”
“I really appreciated having access to the Juntos program, it made a big difference in several of my patients' lives”
“Juntos is such a wonderful service and I'm very thankful that JHH thought to do this in such a timely manner”
“Juntos program was invaluable in ensuring the patient's family had a complete understanding of [the patient's] degree of illness and poor trajectory… I strongly believe the Juntos program should become a permanent part of the JHH language service team as they were vital in facilitating very complicated and nuanced conversations.”

JHH, Johns Hopkins Hospital.

A total of 13 of the 16 Juntos volunteers completed a satisfaction and feedback survey (response rate 81%). The majority indicated that they were either very satisfied (11/16, 71%) or satisfied (2/16, 14%) with their experience. Volunteers indicated that they would be willing to continue to support the program long term if they received organizational recognition, billable time, weekend/moonlight pay, and there was clear support for the future goals and development of the program and the volunteer's career trajectory. All respondents indicated that the role should be staffed by a clinician.

## Discussion

In light of an unprecedented number of Latino patients with LEP admitted at JHH and JHBMC for COVID-19, the Juntos consult service was established to assist with literacy level culturally appropriate and linguistically congruent communication. Between May 5, 2020 to July 30, 2020, 19 bilingual health care providers staffed Juntos consults, including attending physicians, trainee physicians, an advanced practice clinician, registered nurses, and social workers. During this period, a total of 127 Latino patients with LEP were seen in different hospital settings (e.g., medical intensive care unit, general medicine and surgical floors, and labor and delivery). The program offered critical support tailored to the patients' and primary teams' needs (e.g., education, family engagement, goals of care, mental status assessment), and facilitated discharge planning for a patient population otherwise without access to many safety-net benefits. During this time, primary teams requesting the Juntos consult service's assistance expressed the value of the program and elevation of the standards of care for their patients.

Numerous reports indicate Latinos being affected disproportionally by COVID-19, leading to adaptation of existing prevention programs and social media initiatives to respond to Latino's needs.^14–20^ To our knowledge, this is the first report on a program directed to address language barriers exacerbated by the COVID-19 pandemic. Culture-based communication was essential when engaging patients' families in care discussions, which are burdened with emotional distress due to social distancing and complex medical conditions.

Juntos was an unanticipated experiment implemented in a time of crisis, and several important lessons were learned. The need for this program revealed a shortage of bilingual capacity in our health system. Baltimore City is an emergent Latino settlement area that has experienced rapid growth of the Latino immigrant community over the last two decades.^9,21^ In many service sectors, including health care, however, there has been a lag in establishing an adequate infrastructure and workforce to meet the needs of this community, especially those with LEP.^9^ While language access services are essential to the care of patients with LEP, it is well documented that having a language-congruent provider improves patient communication, satisfaction, patient-centeredness, and trust in the provider.^22–24^ Importantly, patient-provider language congruency is associated with improved clinical outcomes, including in patients with very low socioeconomic status.^25^

A diverse workforce of health professionals is critical to improve patient care and eliminate disparities among racial and ethnic groups, including patients with LEP, in the United States.^26^ Nevertheless, minorities are largely underrepresented in medicine despite national efforts to increase diversity in the health care workforce. Juntos highlighted the benefit and important contributions from having bilingual and bicultural providers, however, relying on volunteers only is an unsustainable response to the deep-seated health disparities uncovered and exacerbated by COVID-19. The long-term strategy to promote health equity will require institutional support, sustained diversity efforts, and appropriate recognition of the contribution of underrepresented minorities in health care.

The Juntos volunteer response during a period of crisis, while laudable and expedient, perpetuated a “minority or cultural tax,” which places the burden of additional formal and informal responsibilities on minority staff, trainees, and faculty to advance institutional diversity, equity, and inclusion goals or initiatives.^27,28^ As hospital operations and academic activities resumed, coverage for Juntos consults became increasingly challenging, and Juntos volunteers were frequently working double shifts, performing their usual responsibilities while covering the Juntos paging channel pager. Competing demands were particularly concerning among the Juntos volunteers who were trainees (residents and fellows) at a critical stage of their career, and consistent with studies showing the extra work burden on minority trainees in acting as “cultural ambassadors.”^29^ While the Juntos team was exclusively composed of providers, nurses and a social worker who willingly volunteered their time, there could be strategies to mitigate the work burden or “minority tax.” For example, formal redeployment of Juntos volunteers to care for Spanish-speaking LEP patients would have relieved them from simultaneous duties and potentially expanded the staffing pool. In addition, providing opportunities for mentorship and research productivity related to this work is important for the career advancement of trainees in academic settings.

In addition to enhancing communication between patients or families and providers, Juntos helped address social determinants of health. The majority (71.6%) of patients served through Juntos did not have a primary care physician, and many had unrecognized or untreated conditions, such as diabetes and obesity, that placed them at risk for severe COVID-19. In addition, the majority worked in low-wage essential jobs, such as construction and the service industry, or they were unemployed and undocumented, leading many to be ineligible for unemployment benefits. A critical aspect of the Juntos strategy was collaborating with the health department and community clinics to connect patients to primary care, food delivery, and the isolation hotel if needed. Collaboration and partnerships with trusted community leaders are essential and require a commitment to invest in their constituents and community at large.^30^

The Juntos program was a critical intervention to address the disproportionate impact of COVID-19 on the Latino community.^1–5^ This experience reinforced the need for cultural-based communication to provide optimal care to Latino patients with LEP. However, this type of program is not sustainable by relying only on underrepresented minorities in health care and highlights the need for our health care system to diversify and appropriately invest in community health. Efforts for this have already begun as a result of our Juntos efforts. According to the 2018 U.S. Census American Community Survey, ∼16 million Latinos living in the U.S. speak English less than “very well.”^31^ Provider-patient language-congruent care is one important step in addressing health disparities among patients with LEP. The Juntos consult service could be a model for providing language-congruent care even beyond COVID-19, especially for complex medical and social cases, but to do so will require institutional investment and rigorous outcomes evaluation.

## Supplementary Material

Supplemental data

## Supplementary Material

Supplementary Data S1

Supplementary Appendix SA1

## Abbreviations Used

EHR: electronic health record
JHBMC: Johns Hopkins Bayview Medical Center
JHH: Johns Hopkins Hospital
JHHS: Johns Hopkins Health System
JHM: Johns Hopkins Medicine
LEP: limited English proficiency
PPE: personal protective equipment
QBS: qualified bilingual staff
