# Supplementary material for: Juntos: A Model for Language Congruent Care to Better Serve Spanish-Speaking Patients with COVID-19
Source: Health Equity. 2021 Dec 8;5(1):826–33. doi: 10.1089/heq.2020.0124 (PMC8742298; doi:10.1089/heq.2020.0124)
Supplement: Supplemental data [file Supp_Data.zip › Juntos Manuscript_JuntosConsultantGuide .docx]

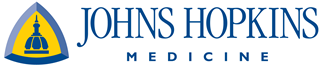


## Juntos Consultant Guide

## Supporting Latinx Patients in the Inpatient Setting

# May 2020 Johns Hopkins Medicine

# *Juntos* *Charter*

# MISSION STATEMENT

# In a collaborative effort between the Johns Hopkins Medicine Office of Diversity and Inclusion, JHM Language Services, and Centro SOL, a program — called *Juntos*— was established on May 5, 2020 to help provide equitable resources and strategies to our Spanish-speaking patients admitted to the hospital with COVID-19.

# STATEMENT OF PROBLEM

# JHM clinicians have identified limited English-proficient (LEP) Latinx patients as a vulnerable group at high risk for COVID-19 infection and late presentation to care. Communication with these patients in the hospital is complicated by the use of personal protective equipment (PPE), as well as other factors, such as fear of deportation and stigmatization, along with additional social concerns (e.g., housing insecurity, crowded living and/or work conditions, and the need to work due to lack of safety net benefits).

# GOAL

# The overall goal of this intervention is to improve the communication with LEP Latinx patients diagnosed with COVID-19, by deploying bilingual and culturally competent clinicians who can work together (*Juntos*) with the primary team to optimize clinical care communication, engage family members as appropriate, and address relevant issues that may impact recovery and safe discharge.

# ROLES AND RESPONSIBILITIES

# The providers working in this role will work synergistically with the medical, clinical and social work teams to explain the treatment plan to the patient and relatives, explore the patient’s social context, and facilitate post-discharge care. The overall goals are to: a) Promote language- congruent care with cultural sensitivity; b) Identify barriers to safe discharge, with attention to issues of public health concern (such as shared housing or crowded working conditions); and c) Advocate and facilitate the appropriate resources for these patients to support them and their communities.

#

# *Juntos means “together” in Spanish. We are all in this together.*

# CONTENTS

# Getting Started…………………………………………………………..page 2

# Provider Tools...………………………………………………………….page 3

# Consultant Workflow...……………………………………………….page 4

# Elements of a Juntos Consult by Clinical Site……………….page 5

# Discharge Planning Resources……………………………………pages 6-9

# Palliative Care…………………………………………………………….page 10

# Patient Billing Questions & Financial Assistance…………page 11

# Juntos infographic………………………………………………………page 12

GETTING STARTED

# Juntos consults are currently available to COVID-19 inpatient units at The Johns Hopkins Hospital and Johns Hopkins Bayview Medical Center, as well as to Labor and Delivery patients.

# Juntos Consultation Shifts - Onsite 8 a.m. to 5 p.m. - 7 days a week, and available by phone evenings and nights to assist with remote support, if needed. Providers are encouraged to request consults before noon for same day service.

# The consultant is not on-call to come to the hospital urgently. Juntos is not billed. Juntos is not for interpreting. Juntos is not for emergencies.

# PROVIDER RESPONSIBILITIES

# Assist clinical team to explain treatment plan, procedures, obtain consent, communicate with relatives and clarify questions/concerns for the patient or family.

# Collaborate with social work to address safe discharge. Identify barriers to safe discharge, with attention to issues of public health concern (such as shared housing or crowded working conditions)

# Promote language-congruent care with cultural sensitivity; Promote the effective use of interpretation modalities and collaborate with medical interpreter to coordinate visits with the clinical team, patient and family, if needed.

# Advocate and facilitate the appropriate resources for these patients to support them and their communities.

PROVIDER TOOLS

# JUNTOS CORUS CHANNEL Channel:  *________* Password: *________* - Log into CORUS  - Search for the Juntos channel and select it - Above the "Join" button is a box that says "Enter Admin Password”: *________* - Click on the "Join" button   - When you want to remove yourself, you can go to “i” on the top right corner and click “Leave” the room

# JUNTOS EPIC NOTES & SMART PHRASES

# Create new note -> Type: Note Specialty: Juntos  Notes: brief summary of reason for consult, and salient discussion points. Anything that may be helpful for the team and social worker

- Juntos smartphrases with common resources are available
- Juntos patient lists in EPIC can be shared with covering providers

# CONSULTANT WORKFLOW

#
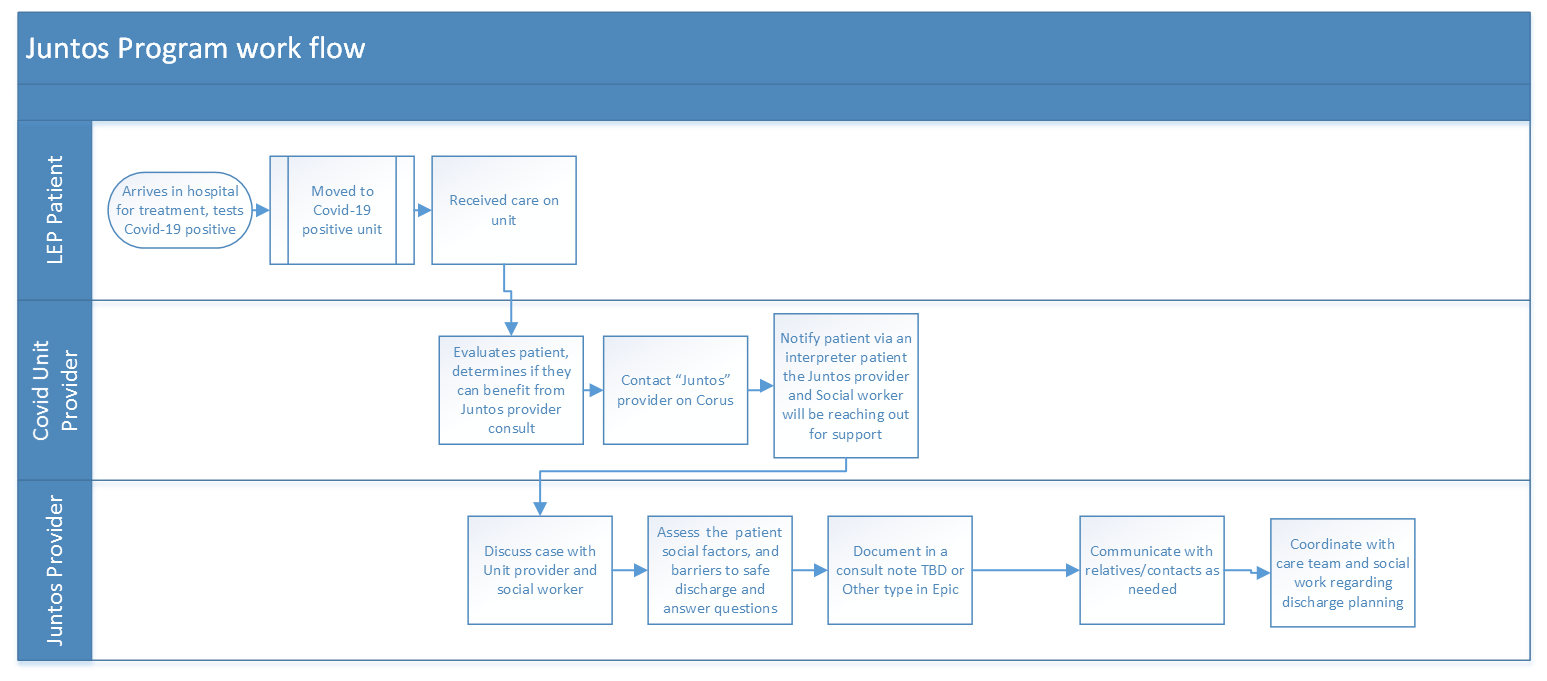


# DETAILED OVERVIEW

# **REVIEW CHART AND DISCUSS WITH PRIMARY TEAM** prior to visiting the patient, the consultant will review the chart and discuss the case with the primary team. It is OK to focus on complex cases and check-in with less complex patients.

# **VIISIT PATIENTS** in person or by video to assess the patient’s understanding of their situation; communicate the treatment plan, address questions/concerns, provide education; answer medical questions; and identify social factors that may impact safe discharge plans (e.g., crowded housing or work conditions, access to food.)

# **COMMUNICATE WITH SOCIAL WORK** to plan for safe discharge.

# **REACH OUT TO PATIENT AND PATIENT’S RELATIVES/CONTACTS** as needed or as requested by the patient and team, including for household COVID-19 testing needs. Consultant will be available for specific team requests, such as follow-up with the family, specific patient teaching, etc.

# **REVIEW PATIENT’S RELATIVES/CONTACTS need for Covid testing** Consultant will assess a patient’s household risk for COVID-19 and consider placing Epic COVID-19 testing orders for household members who have not received testing, or referring to the JHM COVID-19 Hotline.

# Collect family member names, dates of birth and current phone numbers

# Check to see if individuals have Epic MRNs and if not, click “New” to create an MRN in the “Chart” Window in Epic. Place COVID-19 test orders.

# **DOCUMENT JUNTOS NOTES IN EPIC** regarding the patient. A Brief summary of reason for consult, and salient discussion points. Anything that may be helpful for the team and social worker.

# ELEMENTS OF A JUNTOS CONSULT BY CLINICAL SITE

| Labor and Delivery | Floor Consults |
| --- | --- |
| - Education about COVID-19 - Assess mental health (stress, depression, IPV) - Empower mom for joint decision making re: infant   separation - Assess home situation (if separating baby, is there an   uninfected adult?) - Consider ordering test for partner - Assess isolation needs (food, isolation kits) - Explain discharge instructions including isolation   practices and time - Recommend MyChart enrollment - Provide resources for PCP for mom (if she has none)   and other community resources as needed - Provide links to relevant educational graphics/videos - Recommend referral to CART   clinic email for COVID-19 follow up - Explain that she may get call from the health   department for contact tracing and what that means - Can be done in person or remotely - Can be done by non-clinician | - Education about COVID-19 - Assess mental health (stress, depression, IPV) - Assess home situation and feasibility for isolation - Assess isolation needs (food, isolation kits) - Explain discharge instructions including isolation   practices and time - Assess occupation and discuss recs for return to work   (patients may be concerned about medical bills, explain  charity care) - If going to field hospital, assess patient’s understanding  and answer questions (shared bathroom, excellent   provider to patient ratio, temporary - can leave) - Explain CART or PAC follow up if planned - Provide PCP and other community resources if needed - Provide links to relevant educational graphics/videos - Recommend MyChart enrollment - Explain that may get call from health department on   contact tracing and what that means - Can be done in person or remotely - Can be done by non-clinician |
| Pre-Intubation | Post-Extubation |
| - If possible, explain clinical situation, clarify rationale for   treatment (pronation, IL-6 inhibitor, immunoglobulins,   etc.) - If possible, discuss goals of care, clarify code status,   emergency contact, etc. - In person visit, if feasible, is helpful - Update family - Ask team to Corus with updates if they want   ongoing Juntos involvement | - Patient often sedated, delirious, disoriented- our goal is to help reorient and explain what happened and next steps - Engaging family is essential- coordinate with nursing to use iPad for family video visits, can use JH International for out of country calls, try to be there for the first call so you prepare the family to see their critically ill loved one and explain that at first, it’ll likely be a one way but meaningful encounter - Assess mental health - In person visit, if feasible, is helpful - Engage chaplaincy if indicated - Initial home assessment in preparation for discharge   (depending on length of ICU, some people need   rehab) - Some patients are worried about work, medical bills - Ask team to Corus with updates if they want   ongoing Juntos involvement |
| Intubated | End of Life |
| - Mostly family updates - Can organize with nursing that family calls in so patient   can hear voice (very meaningful to family) - Can be done remotely - If there is prognostic uncertainty or family   conflict recommend palliative care consult - Ask team to update through Juntos Corus if they want   us to call family on an ongoing basis | - Family involvement for goal setting- language   concordance and cultural understanding very helpful - If terminal, family can visit once- if possible, excellent to meet family, prepare them for the visit, and enter the rooms with them - Recommend palliative care consult - Chaplaincy if appropriate - Explain to family how decedent services function and   funeral arrangements - A lot (except meeting family if visiting) can be done   remotely |

# DISCHARGE PLANNING RESOURCES

JHM Post-Covid Clinics

| JHH | CART for positive tests already in the system; Patients followed by phone while they are Covid+ |
| --- | --- |
| Johns Hopkins Bayview Medical Center | Pulmonary PACT for those post ICU intubation all mental and physical conditions of the patient to assure a multi-disciplinary evaluation is undertaken and resources are provided |
| Bayview COVID-19 Response Outpatient Clinics | COVID-19+ result within the previous 14 days from time of appointment request  Urgent needs only |

COVID-19 ISOLATION HOUSING

| HOUSING |  |  |  |
| --- | --- | --- | --- |
| BALTIMORE CITY ISOLATION HOTEL | The Lord Baltimore Hotel 20 West Baltimore Street, Baltimore, MD 21201 | - Must be waiting on COVID-19 test results, OR Have a confirmed positive test result - Must agree to take medical transport to the facility - Must meet housing, clinical and safety criteria - **Do not have a home OR** Cannot separate themselves from others in the household who may be at risk for COVID-19. Families are accepted - **Service providers** can call  301-957-1819 - **Patients can call** 443-984-8915 | 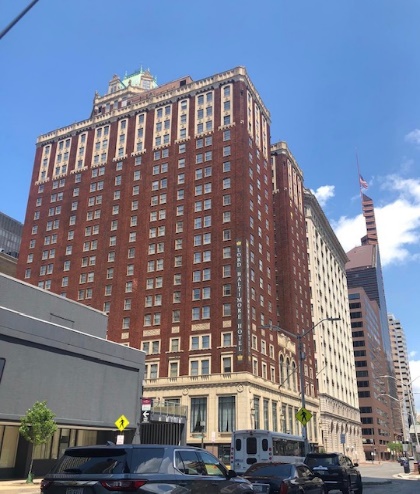 |

COMMUNITY-BASED PRIMARY CARE CLINICS ACCEPTING NEW PATIENTS

**BALTIMORE AREA**

**Healthcare for the Homeless** <https://www.hchmd.org/>421 Fallsway Road, 21202
MON, TUE, WED & FRI 7:30AM – 5:00P, THUR 7:30AM – 12:00PM
FOR APPOINTMENTS: 410-837-5533
Please fax discharge summaries to
443-783-9241 
*Broad definition of homelessness that includes unstable living situations, crowded housing, etc.

**Chase Brexton Health Care** <https://www.chasebrexton.org/> 
Baltimore City (Mt Vernon 1111 N. Charles St. 21201), Baltimore County and Howard County locations
MON – FRI 8:00AM – 5:00PM
FOR APPOINTMENTS: 443-573-5045 
*Please mention “Juntos” when calling

**The Esperanza Center**
430 S. Broadway, 21231
MON – FRI 8:30 am – 4:30 pm 
LIMITED CAPACITY. FOR APPOINTMENTS: 667-600-2919

**PRINCE GEORGE’S AND MONTGOMERY COUNTIES**

**Mary’s Center** <https://maryscenter.org/> 
DC, PG and Montgomery County locations
HOURS VARY BY LOCATION
FOR APPOINTMENTS: 1-844-796-2797

**La Clinica del Pueblo** <https://lcdp.org>
DC and PG locations
HOURS VARY BY LOCATION
FOR APPOINTMENTS DC: 202-462-4788 / PG: 240-714-5247

**Proyecto Salud** <https://proyectosalud.org>
PG and Montgomery County locations
HOURS VARY BY LOCATION
FOR APPOINTMENTS: PG: 301-260-1073 / Montgomery County 301-962-6173

FOOD ASSISTANCE **- ONLY service providers can request food assistance
DESCRIPTION:** The Baltimore City Health Department has Amazon food box delivery for COVID-19 positive individuals who cannot otherwise safely access food due to social and financial barriers during the pandemic. Providers must complete an order form on behalf of the patient.

ONLY service providers can request food delivery and the order form is private; please do not share publicly**.**

LEGAL SERVICES FOR IMMIGRANTS IN BALTIMORE CITY

- Pro-bono Resource Center of Maryland 443-465-4627, <https://probonomd.org/>
- Women’s Law Center 410-396-3294; <http://www.wlcmd.org/>
- Baltimore City has legal resources available to qualifying immigrants who reside in the city, have financial need, and meet certain requirements. Services offered: Community education, immigration legal services, deportation defense services, and bond representation <https://mima.baltimorecity.gov/safe-city-baltimore>

DOMESTIC VIOLENCE

- **Sexual Assault and Domestic Violence Hotline** – Turnaround 443-279-0379 <https://turnaroundinc.org/>
- **House of Ruth** <https://hruth.org/> Help is available in every language. English or Spanish (410) 889-7884 – Press 8 for Spanish.

MAYOR’S OFFICE OF IMMIGANT AFFAIRS: RESOURCE GUIDE
<https://bit.ly/2MIPAUr>

Resources Include:

- Food
- Legal services
- Rent/Housing
- Physical and mental health
- Domestic violence
- Cash assistance
- Child care
- General social services

FREE VIRTUAL MENTAL HEALTH SUPPORT GROUP

“Testimonios Virtual”

Spanish-language virtual support group facilitated by Johns Hopkins mental health clinicians
Women’s Group: 1^st^ and 3^rd^ Tuesdays of the month at 5:30pm

Mens’ Group: 2^nd^ and 4^th^ Tuesdays at 5:30pm

**INVITATION ONLY**

**Process for Patient Transfer from Originating Facility to Baltimore Convention Center Field Hospital**

1. Physician at originating facility writes orders to discharge Patient to the Baltimore
    Convention Center
2. All transfer should will be initiated through the UMMC Access Center.
3. Access center will maintain patient list with originating hospital of all patients
4. Physician at the originating site will be connected via phone call with a receiving physician and triage nurse to determine if patient meets criteria
5. Physicians discuss if a patient meets the criteria for transfer to BCCFH
6. Physicians jointly agree to admit patient to the BCCFH
7. Access Center reviews Census bed board to confirm capacity for patient
8. BCCFH provider accepts the patient
9. The Originating Physician will initiate documentation and coordination transportation
10. Originating facility Case Management will coordinate with families to pick up extraneous
     items
11. Originating provider will communicate to Case Management and Social Work
12. Patient is discharged from originating facility
13. Patient is triaged to BCCFH
14. Documentation is given to EMS Unit
15. In transfer, the patient should only have the following personal items:
16. Street clothes
17. Identification
18. Med pack provided by hospital with 30 days of medications (preferable)
19. Cell phone and phone charger and other electronic devices (iPad)
20. Reading material
21. Drop off patient at designated BCCFH Drop-Off Location
22. Meet Tier I nurse at Triage
23. Deliver documentation to Tier I nurse
24. EMS Does not go beyond Triage location


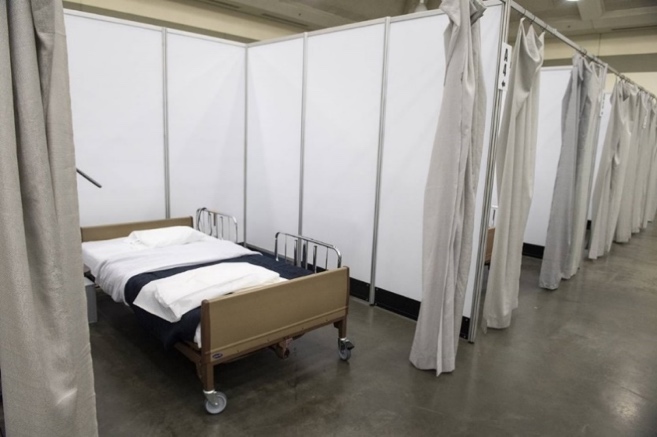


PALLIATIVE CARE

# When should palliative care be involved? When the need has been identified. The comparison between palliative and general provider need is outlined below. Social work is usually involved.

| Palliative Specialty | Palliative Generalist |
| --- | --- |
| Complex intractable symptomsDifficult family conflictsChallenging goals of careComplicated EOL care and decision making, such as withholding or withdrawing medical treatmentsDifficult cases of prognostication/prognostic uncertainty | Management of chronic and progressive disorders with symptom managementCommunication of prognosisBasic pain management, discussions and assistance with emotional and spiritual distressDiscussion about goals of care and code status |

# EDUCATIONAL RESOURCES FOR PROVIDERS AND PATIENTS

# Johns Hopkins Medicine Spanish Resources Portal for COVID-19 <https://www.hopkinsmedicine.org/coronavirus/espanol/index.html>

# Vital Talk: <https://www.vitaltalk.org/wp-content/uploads/VitalTalk_COVID_Spanish.pdf>

# Prepare for your Care: <https://prepareforyourcare.org/content/default/common/documents/PREPARE-COVID-19-Tip-Sheet-National-Spanish.pdf>

Serious Illness Conversation: <https://covid19.ariadnelabs.org/serious-illness-care-program-covid-19-response-toolkit/#outpatient-resources>

PATIENT BILLING QUESTIONS & FINANCIAL ASSISTANCE PROGRAMS

# **Assistance to cover Emergency Admissions** Hospital admissions that result from an Emergency Department visit are billed to a special program managed by the Maryland Department of Health called “Emergency Medicaid”. Hospitals have 90 days to submit Emergency Medicaid applications for reimbursement. Potentially eligible patients are engaged prior to discharge by the hospital’s Medicaid Advocacy team. Johns Hopkins contracts with outside vendors to help with applications. Patients will receive bills for their hospital admissions and should be encouraged to answer the phone and participate in the application process, which is usually initiated by phone before discharge. There is no risk in applying and the hospital does not share any information with immigration authorities. While applications are in process, patients will receive statements by mail.

# **Follow up specialty and primary care: TAP - The Access Partnership** Undocumented, uninsurable patients with demonstrated financial need can be eligible for financial assistance through the TAP program to cover medically necessary outpatient specialty care at JHH and JHBMC. Patients must live permanently in Maryland, have no access to health insurance and be at or below 200% of the Federal Poverty Level. All specialty referrals are reviewed for medical necessity. The TAP program partners with Johns Hopkins Medicine clinics, as well as several community-based clinics where patients can be assisted with the enrollment process by a bilingual TAP navigator.

*As of May 2020, some clinics are not currently accepting new patients due to capacity.
Please verify before referring.*

| Johns Hopkins Clinics | Baltimore Community Clinics |
| --- | --- |
| East Baltimore Medical Center (EBMC)   410-502-8408  1000 E. Eager Street  Baltimore, MD 21202   John Hopkins Outpatient Center (JHOC) Adult Medicine Clinic  410 955-6634  601 N. Caroline Street – 7th Floor   Baltimore, MD 21287  JHBMC General Internal Medicine Clinic & The Children’s Medical Practice  410-550-5511 or 410-550-8847  4940 Eastern Avenue – 301 Building   Baltimore, MD 21224 | **The Esperanza Center**  443-825-3435  430 S. Broadway  Baltimore, MD 21231  **Healthcare for the Homeless**  443-703-1148  421 Fallsway Road  Baltimore, MD 21202    **Baltimore Medical Systems**  410-558-4943  Highlandtown: 3700 Fleet Street, Suite 200    **Chase Brexton Health Services**  410-837-2050 ext. 1205 |

JUNTOS INFOGRAPHIC

*Touch-base with the patient. Ensure he/she understands what is happening. Help elicit history and encourage questions.*

*Engage the family for goal setting. Consult Palliative Care and chaplaincy, if indicated. Explain how decedent services function and communicate about funeral arrangements. Language and cultural sensitivity are crucial for end of life discussions.*

*Keep the family informed to alleviate stress and fear. Promote access to COVID-19 testing.*

*Many family meetings can be done virtually. International calls can be made using tablets and are meaningful. Family can visit once if the patient is terminal.*

*Patients may be concerned about medical bills. Explain charity care and CARE Act Coverage.*

*Helpful for patients in the hospital and their families. Stress related to disease, income loss and medical bills.*

*Explain discharge plan and consult with social work. The patient may need food at home and primary care follow-up.*
