# Supplementary material for: Juntos: A Model for Language Congruent Care to Better Serve Spanish-Speaking Patients with COVID-19
Source: Health Equity. 2021 Dec 8;5(1):826–33. doi: 10.1089/heq.2020.0124 (PMC8742298; doi:10.1089/heq.2020.0124)
Supplement: Supplemental data [file Supp_Data.zip › Juntos Manuscript_IRB.pdf]

JOHNS HOPKINS
MEDICINE

Office of Human Subjects Research
Institutional Review Boards

1620 McElderry Street, Reed Hall, Suite B-130
Baltimore, Maryland 21205-1911
410-955-3008
410-955-4367 Fax
e-mail: jhmeirb@jhmi.edu

Date: August 11, 2020

APPLICATION ACKNOWLEDGEMENT

**Review Type:**	Not Human Subjects Research (NHSR)/Quality Improvement (QI)
**Principal Investigator:**	Kathleen Page
**Number:**	IRB00252774
**Title:**	Covid-19 response for the Latino community
**Committee Chair:**	Richard Moore
**IRB Committee:**	IRB-3

Date of acknowledgement: August 10, 2020

Date of expiration: August 10, 2023

The JHM IRB has determined that the above-referenced new application does not constitute human subjects research under the DHHS or FDA regulations. You may proceed with this project without further interaction with the JHM IRB. If there are changes in this project that may affect this determination, you should consult with the JHM IRB before making those changes.

To keep the JHM IRB application current we are assigning an Expiration Date as noted above. Prior to the expiration date, you will receive an email notification indicating that some action is required. If the Board has determined that a Continuing Review or Progress Report is required, you will need to submit Continuing Review or Progress Report prior to the expiration date. If the Board has determined that No Progress Report is required, you may run the administrative extend approval function.

Expiration Date: The expiration date for this research is listed above. If a continuing review application is required for this research and the approval lapses, the research must stop and you must submit a request to the IRB to determine whether it is in the best interests of individual participants to continue with protocol-related procedures.

Continuing Review/Progress Report: Continuing Review/Progress Report Applications should be submitted at least 6 weeks prior to the study expiration date.

If a progress report is required, failure to submit a progress report in the time period requested will result in your inability to submit any further study actions other than a progress report until your progress report is submitted and acknowledged.

If a Continuing Review application is required, failure to allow sufficient time for review may

result in a lapse of approval. If the Continuing Review Application is not submitted prior to the expiration date, your study will be terminated and a New Application must be submitted to reinitiate the research.

Unanticipated Problems: All unanticipated problems must be submitted using a Protocol Event Report.

If this research has a commercial sponsor, the research may not start until the sponsor and JHU have signed a contract.

Study documents:

Additional Supplemental Study Documents:
ICCTR approval.docx
Resulting Database
Data Collection Questions in Redcap

Protocol:
Latino_eformQ.clean.docx
